# Development of a multiplex assay to assess activated p300/CBP in circulating prostate tumor cells

**DOI:** 10.18632/oncotarget.28477

**Published:** 2023-07-20

**Authors:** Mikolaj Filon, Bing Yang, Tanaya A. Purohit, Jennifer Schehr, Anupama Singh, Marcelo Bigarella, Peter Lewis, John Denu, Joshua Lang, David F. Jarrard

**Affiliations:** ^1^Department of Urology, School of Medicine and Public Health, University of Wisconsin, Madison, WI 53705, USA; ^2^Department of Hematology/Oncology, University of Wisconsin, Madison, WI 53705, USA; ^3^Biomolecular Chemistry, University of Wisconsin, Madison, WI 53705, USA; ^4^Carbone Comprehensive Cancer Center, University of Wisconsin, Madison, WI 53705, USA

**Keywords:** prostate cancer, circulating tumor cells, p300/CBP

## Abstract

Reduced SIRT2 deacetylation and increased p300 acetylation activity leads to a concerted mechanism of hyperacetylation at specific histone lysine sites (H3K9, H3K14, and H3K18) in castration-resistant prostate cancer (CRPC). We examined whether circulating tumor cells (CTCs) identify patients with altered p300/CBP acetylation. CTCs were isolated from 13 advanced PC patients using Exclusion-based Sample Preparation (ESP) technology. Bound cells underwent immunofluorescent staining for histone modifying enzymes (HMEs) of interest and image capture with NIS-Elements software. Using the cBioPortal PCF/SU2C dataset, the response of CRPC to androgen receptor signaling inhibitors (ARSI) was analyzed in 50 subjects. Staining optimization and specificity revealed clear expression of acetyl-p300, acetyl-H3K18, and SIRT2 on CTCs (CK positive, CD45 negative cells). Exposure to A-485, a selective p300/CBP catalytic inhibitor, reduced p300 and H3K18 acetylation. In CRPC patients, a-p300 strongly correlated with its target acetylated H3k18 (Pearson’s R = 0.61), and SIRT2 expression showed robust negative correlation with a-H3k18 (R = −0.60). A subgroup of CRPC patients (6/11; 55%) demonstrated consistent upregulation of acetylation based on these markers. To examine the clinical impact of upregulation of the CBP/p300 axis, CRPC patients with reduced deacetylase SIRT2 expression demonstrate shorter response times to ARSI therapy (5.9 vs. 12 mo; *p* = 0.03). A subset of CRPC patients demonstrate increased p300/CBP activity based on a novel CTC biomarker assay. With further development, this biomarker suite may be used to identify candidates for CBP/p300 acetylation inhibitors in clinical development.

## INTRODUCTION

Androgen deprivation therapy (ADT) is the cornerstone treatment for advanced PC, but most patients develop castration-resistant prostate cancer (CRPC) 36-40 months after initiating ADT [1]. Additional therapies have been developed, but novel targets are needed. The variation in effectiveness of many of these drugs and considerable side effects of systemic administration impact the care of patients with advanced PC. Being able to leverage drug therapy specifically based on molecular susceptibilities that advanced cancers exhibit provides an attractive approach to personalize patient treatment.

Histone modifying enzymes (HMEs) result in histone post-translational modifications that alter gene expression and cell behavior [2]. HMEs make attractive drug targets due to their enzymatic activity and specific dysregulation in certain cancers. P300, is a transcriptional co-activator which functions as a histone acetyltransferase by modulating chromatin [3–5]. Its enzymatic activity is opposed by Sirtuin 2 (SIRT2) that negatively regulates the acetyltransferase activity of p300 via deacetylation of an automodification loop within its catalytic domain [6]. Our group recently discovered using a novel chip-based histone enzyme screen that increased acetylation activity of p300 occurs during the development of hormone-resistant PC in a subset of CRPC tumors [2]. Reduced SIRT2 expression and increased p300 activity leads to a concerted mechanism of hyperacetylation at specific histone lysine sites (H3K9, H3K14, and H3K18) in CRPC tissues altering gene expression. A dependency of H3K18 acetylation on p300 activity was confirmed in PC cells [2]. More recent work has confirmed these findings in CRPC [7]. A suite of CBP/p300 inhibitors are under clinical evaluation for a number of solid and hematologic tumors [8]. These findings have generated interest in determining whether P300, SIRT2 and their targets act as biomarkers to identify patient tumors with increased p300 acetylation activity for personalized drug treatment.

Circulating tumor cells (CTCs) have generated interest as minimally invasive, biomarkers for cancer management [9, 10]. CTCs separate from a primary tumor or metastatic sites to enter the bloodstream. Researchers have reported that following treatment, CTC enumeration in prostate and breast cancer patients (based on EpCAM-captured CTCs) has prognostic potential in informing treatment outcomes [11–13]. Circulating tumor cell-based measurement of AR-V7 is positively associated with primary resistance to abiraterone and enzalutamide in CRPC [14]. These findings make CTCs a uniquely poised asset with which to monitor response to anti-cancer therapies, guide treatment, and make an accurate prognosis.

In the current study, employing a novel Exclusion-based Sample Preparation (ESP) technology (9) (Supplementary Figure 1) to isolate CTCs, we evaluated p300 activity, SIRT2 expression and H3K18 acetylation in CTCs in a series of patients with sensitivity or resistance to ADT. We find a concerted increase in the expression of H3k18 acetylation and acetyl–p300 and downregulation of SIRT2 in CTCs in a subset of CRPC patients. Decreased SIRT2 is associated with shorter time of response to ARSIs. The results from this initial cohort support the integration of these biomarkers into prospective clinical trials.

## RESULTS

### Optimization of the p300 CTC assay

**Figure 1:**
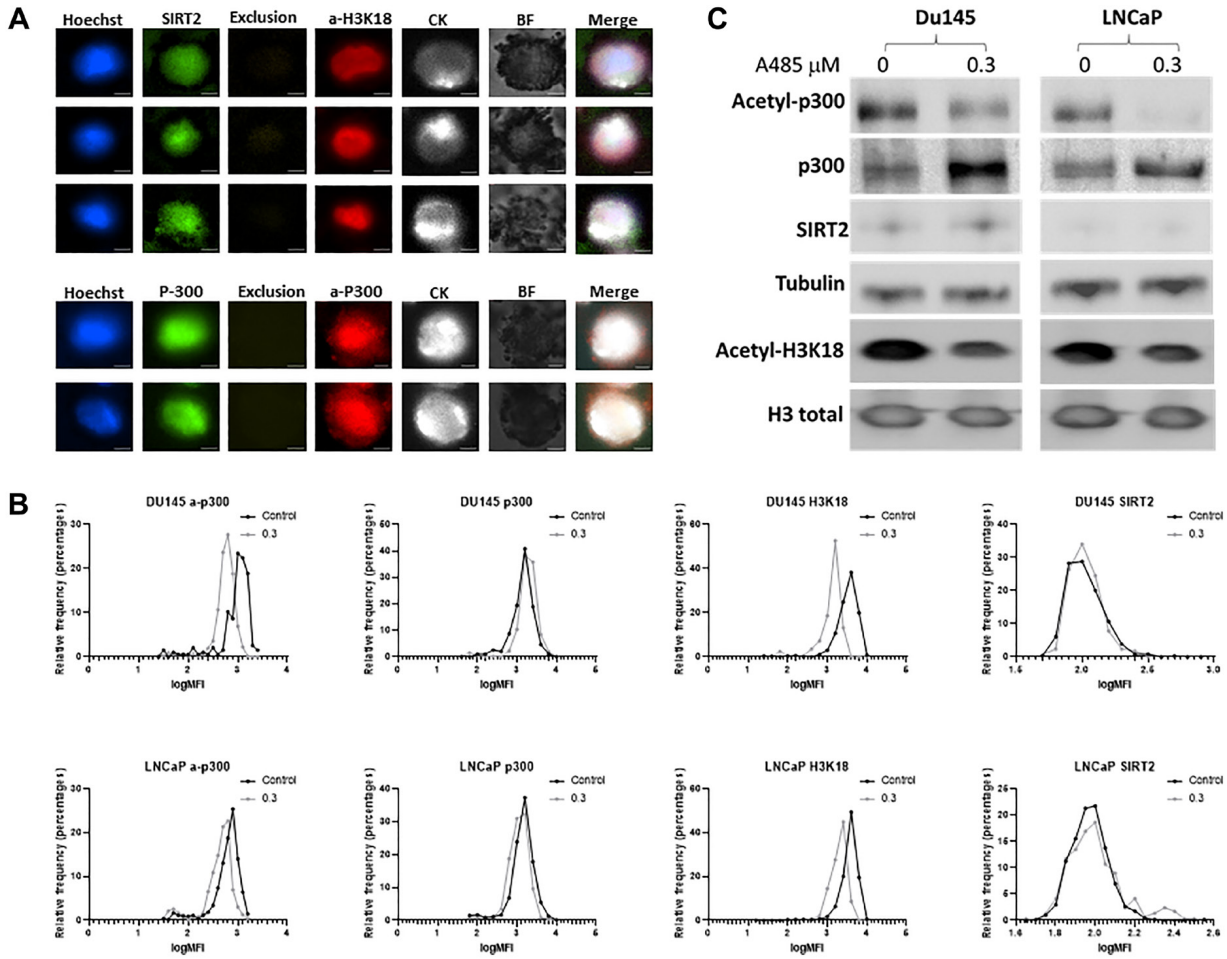
Optimization of circulating tumor cells (CTC) staining and validation of p300 activity. Prostate cancer cell lines LNCaP and Du145 cells were spiked into whole blood and captured using an EpCAM antibody and stained with acetyl-p300, total p300, acetyl-H3K18 and SIRT2 epigenetic biomarkers Cells of exclusion (not captured with EpCAM) being stained with CD11b, CD66b, CD45 and CD34 showed no positive staining. (**A**) Cells were stained (LNCaP shown) and imaged with a fluorescence microscope (Magnification X200). Cytokeratin (CK) is used to identify intact cells and Hoeschst staining employed for evaluating cell nuclei. (**B**) Cells were treated with 0.3 μM of A-485 for 24 hours then harvested for staining and analysis. A-485 is a selective catalytic inhibitor for p300 histone acetyltransferase. Staining for each channel is demonstrated. A reduction in acetyl-P300 and acetyl-H3K18 (left shift) is noted after inhibitor treatment. Assays were performed in duplicate with excellent reproducibility. (**C**) Western blot confirms the reduction in acetylated p300 and its target, acetylated H3k18 by A-485 seen on fluorescent staining. Experiments were repeated and performed in duplicate.

Previously validated antibodies against acetyl-p300, total p300, SIRT2 and acetyl-H3k18 were tested in single-cell staining assays using LNCaP and Du145 initially (Figure 1A). Positive staining for cytokeratin confirms intact cells and Hoechst identifies cell nuclear staining, with acetyl-p300, total p300, SIRT2 and acetyl- H3k18 signals each optimized at their specific fluorescence. Exclusion approaches previously described [15] using CD66b, CD45 and CD34 show no staining and are intended to differentiate neutrophil, monocyte, macrophage, or lymphocytes from CTCs when patient blood samples are evaluated. Bright field confirmed the absence of a background signal.

We previously demonstrated that acetyl-p300, acetyl-H3K18, and SIRT2 reflect p300 acetylation enzyme activity in PC cells [2]. To examine this in the current exclusion assay, we compared the expression in the hormone-resistant cell line Du145 and hormone-sensitive LNCaP after treatment with a selective p300 inhibitor (A-485). A-485 is a catalytic inhibitor of the p300 histone acetyltransferase [16]. The two cell lines were exposed to 0.3 μM of A-485 for 24 hours, then half of the cells were used for the CTC assay and the rest to check the protein expression for acetyl-p300, total p300 and acetyl-H3K18 using western blot. After A-485 treatment, the cellular level of p300 acetylation was reduced in both Du145 and LNCaP cells as evidenced by a reduction in fluorescence (Figure 1B). LNCaP cells express lower levels of basal p300 acetylation activity [2]. Reduction of p300 acetylation protein in Du145 cells (54%) and LNCaP cells (91%) is confirmed in conjunction with a compensatory increase in un-acetylated p300 by western blot (Figure 1C). Decreases are also observed in H3k18 acetylation, a target of CBP/p300 in both cell lines. SIRT2 is not altered with this specific p300 acetylation inhibitor.

**Figure 2:**
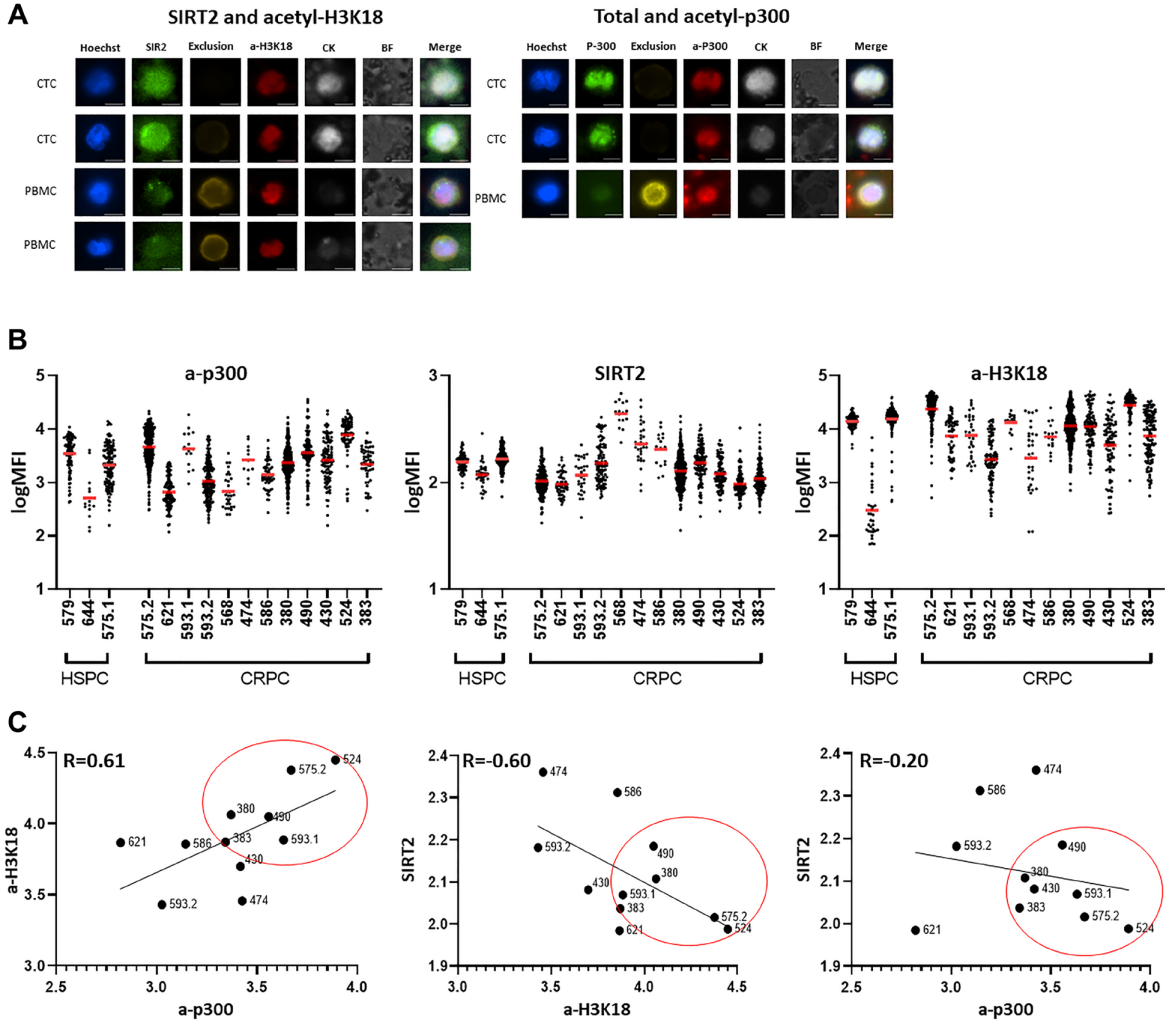
Staining on CTCs in PC patients identifies a subset with increased acetyl-p300 and H3K18 and reduced levels of the deacetylase SIRT2. (**A**) Peripheral blood mononuclear cells (PBMCs) were isolated from patient blood samples with Ficoll-Paque PLUS then fixed with Cytofix. Fixed PBMCs were captured via EpCAM to isolate CTCs and then stained with the target biomarkers. CTCs were successfully captured by EpCAM antibody and show clear expression of acetyl-p300 (a-p300), acetyl-H3k18 (a-H3K18), SIRT2 and CK (cytokeratin), and no expression with exclusion staining. In contrast, lack of staining for CK but bright staining for exclusion was shown in the PBMC (control), confirming that both CTC isolation and staining were successful. (**B**) Levels of biomarkers in CTC sets from individual patients. The three samples on the left of the graph are from HSPC patients, and the right from CRPC patients. There was a trend of increased acetylation of a-p300 and a- H3K18, and decreased SIRT2 with CRPC development directly compared in one patient (#575). Patient #593 got a 2nd test after beginning enzalutamide. (**C**) In CTCs from CRPC, a-p300 shows a strong positive correlation with its target a-H3k18 histone PTM (R = 0.61, *p* = 0.0443, left panel). SIRT2 expression, a deacetylase, demonstrates a robust negative correlation with a-H3k18 (R = −0.60, *p* = 0.05; middle panel), and a-p300 showed a negative correlation with SIRT2 (R = −0.2, *p* = 0.55; right panel). These biomarkers are consistently altered in a subset of patients (#524,575.2,490,380,593.1,380,383) indicating increased acetylation activity in these samples.

The epigenetic biomarker suite was initially optimized on CTCs generated from several patients. Patient PBMCs (blood monocytes/mononuclear cells) were isolated from blood samples, captured via EpCAM, and then stained with the target biomarkers. PBMCs without captured by EpCAM are used as controls to compare expression intensity between CTCs as described [9]. PBMCs demonstrate negative staining for cytokeratin, but stain positively for exclusion markers CD66b, CD45 and CD34. In contrast, CTCs successfully captured by the EpCAM antibody express acetyl-p300, acetyl-H3k18 and SIRT2, as well as cytokeratin, but lack exclusion staining (Figure 2A).

### Evaluation of p300 acetylation activity staining on CTCs in PC patients

Clinicopathologic characteristics are outlined in Table 1 and consisted of 12 CRPC (castration resistant PC) and 3 HSPC (hormone sensitive PC) samples. One patient (#575) had both hormone-sensitive and subsequent CRPC samples collected. CTCs were captured with the EpCAM antibody and processed as described in the Materials and Methods. Total captured and marker positive CTCs are shown in Table 1. In Figure 2B the staining intensity of each CTC for a-p300, SIRT2 and a-H3K18 with the mean for each patient is presented. Clustering of individual cells around the mean is noted in the majority of patients. Most markers demonstrate some heterogeneity of expression with SIRT2 expression demonstrating the most uniform expression (Supplementary Table 1).

**Table 1:** Clinicopathologic patient information and androgen sensitivity

Hormonal status	ID code	Age	Gleason grade	Lymph node +	Bone metastases	Prior treatments	PSA (ng/ml)	Captured CTC#
HSPC	579	66	4+4 = 8	Yes	High-volume, Bone	None	1204	192
HSPC	644	77	NA	Yes	Node only	None	158	48
HSPC	575.1	70	4+5 = 9	Yes	Low-volume, Bone	None	2.4	304
CRPC	575.2	71	4+5 = 9	No	Low-volume, Bone	LHRHa; Docetaxel	51	610
CRPC	621	81	4+5 = 9	No	Low-volume, Bone	LHRHa	5.1	161
CRPC	593.1	74	NA	No	Low-volume, Bone	LHRHa; DNA vaccine trial	57	47
CRPC	593.2	75	NA	No	Low-volume, Bone	LHRHa; DNA vaccine trial; anti-CTLA-4 monoclonal antibody	NA	259
CRPC	568	69	3+3 = 6	No	High-volume, Bone	LHRHa; Enzalutamide; DNA vaccine trial	40	31
CRPC	474	69	4+5 = 9	No	Node only	LHRHa	4.2	42
CRPC	586	54	4+4 = 8	Yes	Low-volume, Bone	LHRHa; Enzalutamide; Docetaxel	14	69
CRPC	380	75	4+5 = 9	No	High-volume, Bone	LHRHa; sipuleucel-T; Enzalutamide; Docetaxel; TRC 253 8; Radium-223; Cabazitaxel	726	1251
CRPC	490	76	3+3 = 6	No	High-volume, Bone	LHRHa; DNA vaccine trial; abiraterone; Sacituzumab Govitecan; Docetaxel	328	217
CRPC	430	70	4+5 = 9	No	High-volume, Bone	LHRHa; Docetaxel; Cabazitaxel + abiraterone.	11	193
CRPC	524	59	4+3 = 7	Yes	Low-volume, Bone	LHRHa; Abiraterone + prednisone; Enzalutamide	9.3	166
CRPC	383	62	3+5 = 8	Yes	Low-volume, Bone	DNA vaccine trial; orchiectomy; Enzalutamide	19	181

Abbreviations: NA: not available; HSPC: hormone-sensitive prostate cancer; CRPC: Castration-resistant prostate cancer; DNA: Deoxyribonucleic Acid; DNA vaccine trial: Regramostim, DNA vaccine targeting prostatic acid phosphatase; PSA: prostate-specific antigen; CTLA-4: cytotoxic T-lymphocyte–associated antigen 4; TRC 253: Androgen Receptor Antagonist (TRACON Pharmaceuticals); High-volume: visceral metastases or four or more bone lesions with at least one beyond the vertebral bodies and pelvis.

We previously found that decreased SIRT2 expression, increased p300 acetylation activity, and elevated histone H3 hyperacetylation are associated with the transition from hormone-sensitive to CRPC [2]. Sample number did not permit this to be statistically compared. However, this trend is noted when HSPC (#575.1) is directly compared to a later sample (>12 mo) obtained after the development of CRPC (#575.2). In a separate subject, treatment effects consisting of decreased acetyl-p300, acetyl-H3k18, and increased SIRT2 staining is seen several months after beginning enzalutamide in one patient (#593.2).

We then tested whether there was a correlation between p300/CBP markers in the CTCs obtained from individual patients (Figure 2C). Using Pearson’s correlation, as predicted, acetyl-p300 displays a strong positive correlation with its target acetylated H3k18 (R = 0.61), and SIRT2 expression shows a robust negative correlation with H3k18 (R = −0.60). Acetyl-p300 demonstrates a negative correlation with SIRT2 (R = −0.2) in CRPC. Clustering of increased p300/CBP activity markers was noted in a subset of patients as indicated in Figure 2C.

### Low SIRT2 correlates with shorter response to ARSI treatment in CRPC patients

**Figure 3:**
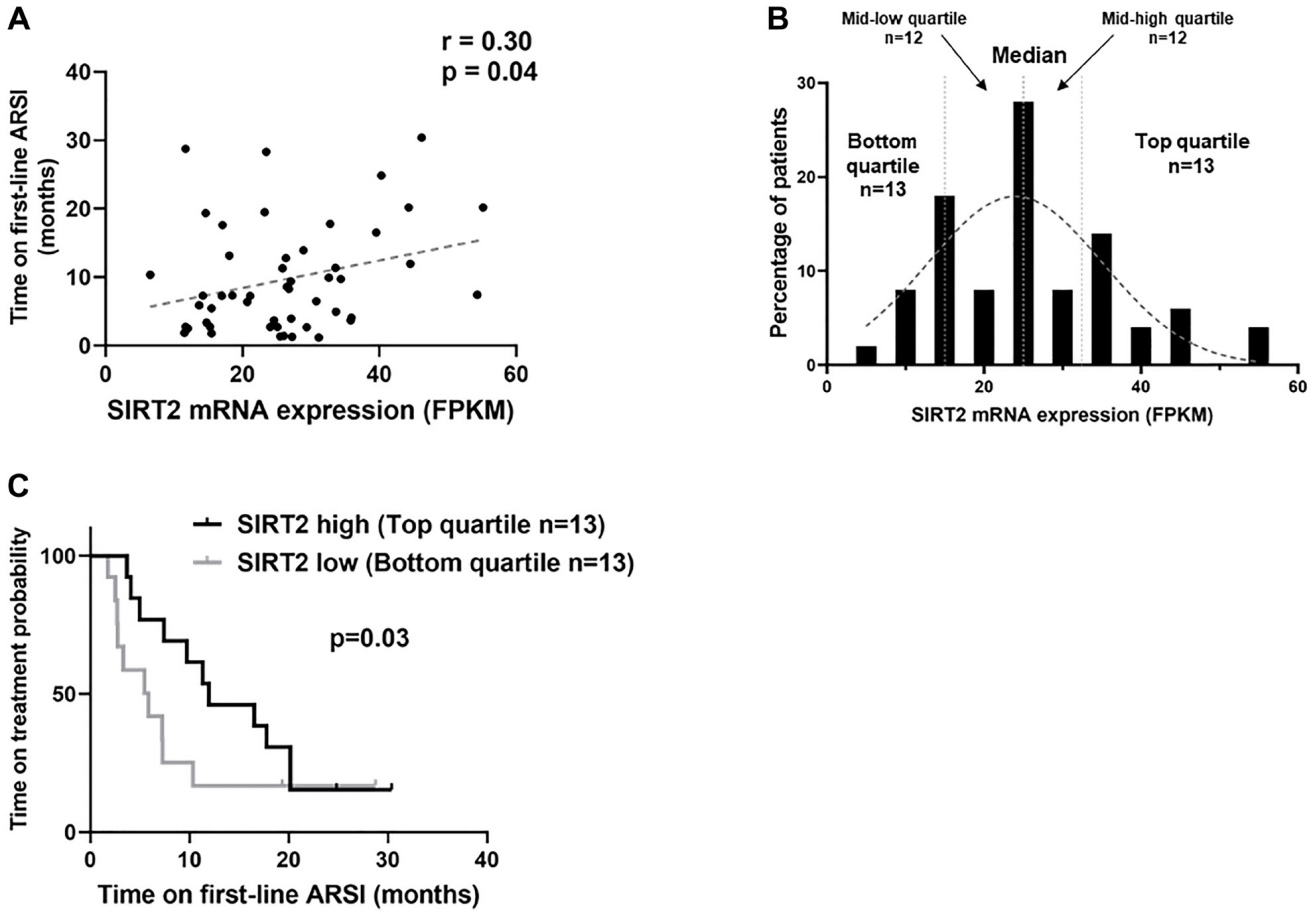
Reduced deacetylase SIRT2 mRNA levels predicts shorter time of response to androgen receptor signaling inhibitors (ARSI). Of 444 CRPC patients in the SU2C/PCF cBioPortal dataset, RNA expression data and clinical outcomes with response to ARSI (abiraterone/enzalutamide) were available for 55 subjects. (**A**) Pearson correlation analysis showed that SIRT2 mRNA levels significantly correlate with progression-free survival time (*p* = 0.04). (**B**) The cohort were divided into quartiles based on the SIRT2 mRNA levels, which revealed a Gaussian-like distribution. (**C**) Kaplan-Meier analysis performed to compare top quartile for SIRT2 expression (*n* = 13) with bottom quartile (*n* = 13). Patients within the lowest SIRT2 expression quartile have a significantly shorter time to progression on ARSIs with SIRT2 high responding 12 months vs. SIRT2 low only 5.9 months (Hazard ratio = 0.53, 95% CI = 0.21 to 1.32, *p* = 0.03).

Given our previous data demonstrating an upregulation of the CBP/p300 pathway in CRPC [2, 17], we further explored its importance by evaluating treatment resistance. First line management of advanced CRPC involves androgen deprivation therapy combined with an androgen signaling inhibitor (ARSI) such as enzalutamide and abiraterone [18]. A recent mCRPC genomic landscape study with linked outcomes data provided an opportunity to evaluate whether activation of CBP/p300 pathway is associated with poorer clinical response to ARSI drugs [18]. Protein acetylation information is not available on cBioPortal, therefore we utilized SIRT2 mRNA expression levels as a surrogate for CBP/P300 activity levels given the known correlation with acetyl-p300 and acetyl-H3K18 (Figure 2C) in CTC, cell lines and tissues [2, 17]. An analysis was performed using clinical outcomes data available on cBioPortal for a well-annotated CRPC dataset (SU2C/PCF *n* = 50). Pearson correlation analysis showed that SIRT2 mRNA levels are significantly correlated with progression-free survival time (R = 0.30, *p* = 0.04) (Figure 3A). To further assess this correlation, we divided the cohort into quartiles based on the SIRT2 mRNA levels, which revealed a Gaussian-like distribution (Figure 3B). We excluded 5 of the 55 patients who had *SPOP* mutations (who were distributed evenly across the quartiles) because these patients have increased sensitivity to abiraterone [19]. Patients with the lowest SIRT2 expression have a significantly shorter time to progression on enzalutamide/abiraterone (SIRT2 high 12 months vs. SIRT2 low 5.9 months; Hazard ratio = 0.53, 95% CI = 0.21 to 1.32, *p* = 0.03) (Figure 3C). This clinical data supports the importance of the CBP/p300-SIRT2 axis in the development and progression of castration resistance.

## DISCUSSION

Although there are multiple available therapies for men with metastatic CRPC, there are currently few molecular biomarkers to help guide optimal treatment choice and predict prognosis in these patients. CTCs have the potential to address this need. The present study is an initial analysis of a biomarker set representing CBP/p300 activity in a series of patient CTCs. We find a strong correlation between p300 acetylation, SIRT2 and its target H3K18 acetylation in patients with advanced CRPC. We also demonstrate in a separate clinical dataset that low SIRT2 levels are associated with a shorter response to drugs that block androgen receptor signaling.

We successfully isolated specific CTCs from patient blood samples, and by quantifying the protein expression in actual CTCs performed with immunocytochemistry (ICC), we found increased p300 activity and H3K18 hyperacetylation, and decreased SIRT2 expression in a subset of patients resistant to ADT treatment (Figure 3). We have previously demonstrated this correlation to be significant in tumor deposits from CRPC where roughly half of the patients demonstrated this pattern (2). Autoacetylation of p300 at K1499 is a target of SIRT2 deacetylase and loss of SIRT2 decreases its inhibition of p300 acetylation. Studies also report that p300 acetylates SIRT2 to attenuate its deacetylation activity forming a feedback loop [20].

P300 activity is critical for the proliferation of PC3 and other CRPC cell lines. Researchers have found that the disruption of p300 transcripts through small interfering RNA inhibited PC cell proliferation both at the basal level and upon interleukin 6 stimulation [21]. In the current CTC investigation, increased P300 activity was confirmed by its increased acetylation of p300 as well as an increase in its target H3K18ac. The relationship between these proteins shown in patients’ CTCs recapitulate the observations found in PC cell lines [2]. A significant positive correlation is shown between acetyl-p300 and acetyl-H3K18 in CRPC patients (Figure 2C). A negative correlation between SIRT2 and acetyl H3k18 and to a lesser extent between acetyl-p300 and SIRT2 was also observed in CRPC patients. The consistent alterations in these markers identifies a set of patients with an increase CBP/p300 activity.

An important insight from our study is how the CBP/p300 axis is associated with treatment response. In one patient, a sample (575.1) was collected when ADT was initiated, and the PSA dropped to 2.4 ng/ml. A second sample (575.2) was obtained one year later when the cancer became androgen independent, and the PSA increased to 50 ng/ml with bone metastases. Acetyl-p300 and H3k18 were higher and SIRT2 was lower in 575.2 (CRPC) than in the responding hormone-sensitive PC sample (575.1). Extending our findings, in a well-annotated clinical database of mCPRC patients, we find that decreased deacetylase SIRT2 expression is associated with a shorter time of response to ARSI (Figure 3). SIRT2 plays an important role in regulating p300 acetylation activity in the development of a subset of CRPC. Autoacetylation of p300 at K1499, a modification known to enhance HAT activity and a target of deacetylation by SIRT2, is highly elevated in CRPC cells, while SIRT2 protein level is reduced [2]. CBP/p300 are potent coactivators of the AR, and perturbation of p300/CBP function in PC models decreases AR function and reduces tumor cell growth [21, 22].

Our study has some limitations. Because of the small sample size, we were unable to achieve a statistical significance comparing HSPC and CRPC groups. CTCs demonstrate heterogeneity (patient-to-patient heterogeneity, and intra-patient CTC variation) [23] and the impact of this on a response to p300 inhibitors is unknown. Confirmation of these findings will require additional studies. Another potential limitation is that undifferentiated prostate cancer cells (i.e., neuroendocrine prostate cancer) may not be captured by EpCAM antibody. Neuroendocrine cancer forms in a small subset (15–20%) of late PCa [24]. EpCAM is the most widely utilized marker to capture the greatest number of circulating PCa cells [9].

The identification of a subset of CRPC patients with increased CBP/p300 activity in CTCs, and previous data demonstrating p300 activity correlates with these three epigenetic biomarkers, suggests they may be used to identify candidates for targeted treatment. P300 inhibitors have been examined in the context of advanced PCa patients, and a recent orally available p300/CBP bromodomain inhibitor (CCS1477) has been found to decrease AR and MYC signaling in PC tissues [7].

## MATERIALS AND METHODS

### Cell culture

The PC cell lines Du145 is resistant to androgen deprivation therapy, and androgen-sensitive LNCaP were obtained from the ATCC and genotyped for validity. Du145 or LNCaP cells were then exposed to A-485 [16], a potent, selective p300/CBP catalytic inhibitor for 24 hours at a dose of 0.3 μM after initial dose ranging studies (0.1–1 μM), Cells were collected for staining with the described biomarkers.

### Patient samples

Peripheral blood was collected after informed written consent from patients with advanced PC as previously described [15] under a University of Wisconsin IRB approved protocol. A maximum of 15 mL of blood was obtained at any given blood draw using EDTA vacutainers (BD). Whole blood was diluted 1:1 with Hank’s balanced salt solution (HBSS, Lonza) and 30 mL of diluted blood was underlaid with 10 mL of Ficoll-Paque PLUS (GE Healthcare) per 50 mL conical tube. The blood was centrifuged for 40 min at 974X g, and resulting buffy coats were washed once with HBSS. Peripheral blood mononuclear cells (PBMCs) were isolated and then fixed with Cytofix.

### Paramagnetic particle (PMP) preparation

Sera-Mag (SM) streptavidin-coated magnetic beads (Cytiva) at a concentration of 50 ug per reaction were employed for all experiments. SMs were washed twice and resuspended in 0.1% Tween-20 (Fisher Scientific) in PBS prior to use. SM solutions were then incubated under agitation for 20 minutes with 1 μL (concentration) of a single capture antibody, against EpCAM (R&D Systems, clone TROP1). Prior to use, bead conjugates were washed an additional three times with 10% FBS.

### CTC isolation

An overview of the CTC capture technology is shown in Supplementary Figure 1. CTCs were captured from PBMCs with antibody labelled SMs. PBMCs were incubated on a rotator with antibody-labelled SMs in a buffer containing 10% FBS for 20 min at 5°C. Cells bound to SMs were isolated with ESP technology using an automated magnetic head attachment for the standard Gilson PipetMax pipetting robot. SM-bound cells were moved through staining and washing wells prior to imaging at 20× with a Nikon Eclipse Ti fluorescent microscope (Nikon) and NIS-Elements AR 4.10 software (Nikon) after a final transfer to glass-bottom chamber slides.

### Fluorescent staining

For all staining, cells were blocked with 10% FBS prior to extracellular staining, then, if intracellularly stained, cells were treated with Permeabilization Solution (BD) and stained for intracellular markers in Perm/Wash Buffer (BD). Stains: Hoechst, and antibodies against CD45 (BioLegend, HI30; Tonbo, HI30), CD34 (BioLegend, 581), CD66b (BioLegend, G10F5), pan-Cytokeratin (pCK, Abcam, C-11), SIRT2 (Proteintech, 66410), Anti-histone H3-acetyl K18 (Abcam, 1191), p300 (Santa Cruz, 32244 AF488), acetyl-p300 (Invitrogen, PA5-16185).

### Image analysis

Images were analyzed with NIS Elements software (Nikon). Average background fluorescence was subtracted from each channel then cells were identified and masked based on user-defined Hoechst staining thresholds, then further evaluated for average cellular staining intensity for all other fluorescent antibodies. Data was output and analyzed in Microsoft Excel, and GraphPad Prism.

Staining intensity is compared between different cells by graphing each cell as an individual data point on a scatter plot, similar to flow cytometry. When graphed in this way, cells distribute into higher and lower density regions, where cells with similar staining characteristics cluster together [25]. These cell clusters are surrounded by low density borders which allow analysts to manually define boundaries with gates. Manual gating is widely accepted in flow cytometry and allows more complex interrogation of staining characteristics on population subsets. In this report, the threshold for positive and negative antibody staining in fluorescence microscopy was based on clustering of cell populations within a representative patient sample. These thresholds were then applied uniformly across all patient samples treated with similar experimental conditions.

### Analysis of cBIOPORTAL prostate cancer database

Data was obtained from a 444 PCF/SU2C metastatic prostate cancer patient cohort (18). RNA-seq data and enzalutamide/abiraterone treatment data were downloaded from cBioPortal (http://www.cbioportal.org/). There were 128 patients in this cohort with metastatic CRPC that have baseline biopsy and matched clinical data and 75 patients of this 128 sub-cohort have gene expression data captured by Poly-A RNA-seq. A subset (55 patients) of this 75 sub-cohort have records of time on either enzalutamide/abiraterone. Five patients of this cohort were excluded because they have *SPOP* mutations that demonstrate elevated sensitivity to antiandrogen treatment [19]. The correlation graph, histogram of *SIRT2* mRNA distribution, and time on treatment graph were generated by GraphPad Prism v9.5.1. The *p*-value for time on treatment probability was calculated using Gehan-Breslow-Wilcoxon test.

## SUPPLEMENTARY MATERIALS



## Abbreviations

ADT: androgen deprivation therapy
CRPC: castration-resistant prostate cancer
CTCs: circulating tumor cells
ESP: exclusion-based sample preparation
HMEs: Histone modifying enzymes
ICC: immunocytochemistry
PC: prostate cancer
SIRT2: Sirtuin 2
ARSI: androgen receptor signaling inhibitors
