# Supplementary material for: Development of a multiplex assay to assess activated p300/CBP in circulating prostate tumor cells
Source: Oncotarget. 2023 Jul 20;14:738–46. doi: 10.18632/oncotarget.28477 (PMC10360924; doi:10.18632/oncotarget.28477)
Supplement: Supplementary file 1 [file oncotarget-14-28477-s001.pdf]

Development of a multiplex assay to assess activated p300/cbp in circulating prostate tumor cells

SUPPLEMENTARY MATERIALS

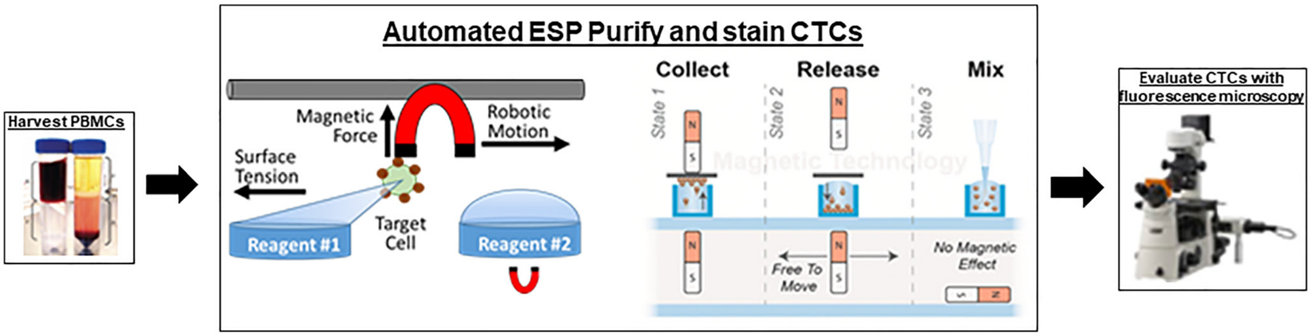

Supplementary Figure 1: Exclusion-based sample processing (ESP) technology enables clinically relevant CTC analytics by maximizing yield and specificity. Antibody-based CTC enrichment is achieved using an automated magnetic head attachment for the standard Gilson PipetMax pipetting robot. Magnetic bead-coated CTCs are gently moved between reagents using surface tension to hold reagents in their respective wells. Magnetic forces above and below the sample enable alternating actions of cell collection and cell release, with controlled pipette mixing for uniformity in sample processing. Commercially available plastic consumables facilitate sterile and high-throughput testing. High-yield CTC enrichment maximizes sensitivity and specificity by reducing the burden of background cells from the circulation and increases the potential for the analyzed population to represent disease.

Supplementary Table 1: Coefficient of variation (CV) for CTC marker staining demonstrates heterogeneity in some markers

*CV	579	644	575.1	575.2	621	593.1	593.2	568	474	586	380	490	430	524	383
a-p300	0.0892	0.1647	0.1137	0.0909	0.1016	0.0957	0.1176	0.1125	0.0860	0.0840	0.0811	0.1127	0.1188	0.0913	0.0980
SIRT2	0.0355	0.0558	0.0390	0.0606	0.0520	0.0743	0.0857	0.0452	0.0791	0.0689	0.0681	0.0734	0.0652	0.0575	0.0691
a-H3K18	0.0297	0.2097	0.0656	0.0659	0.0930	0.1023	0.1224	0.0439	0.1773	0.0684	0.0722	0.0886	0.1402	0.0579	0.1138

*CV: Coefficient of variation.
